# In-Series U-Net Network to 3D Tumor Image Reconstruction for Liver Hepatocellular Carcinoma Recognition

**DOI:** 10.3390/diagnostics11010011

**Published:** 2020-12-23

**Authors:** Wen-Fan Chen, Hsin-You Ou, Keng-Hao Liu, Zhi-Yun Li, Chien-Chang Liao, Shao-Yu Wang, Wen Huang, Yu-Fan Cheng, Cheng-Tang Pan

**Affiliations:** 1 Institute of Medical Science and Technology, National Sun Yat-sen University, Kaohsiung 80424, Taiwan; sallychen@imst.nsysu.edu.tw; 2 Liver Transplantation Program and Departments of Diagnostic Radiology, Kaohsiung Chang Gung Memorial Hospital, Chang Gung University College of Medicine, Kaohsiung 833401, Taiwan; ouhsinyou@gmail.com (H.-Y.O.); liao1009@gmail.com (C.-C.L.); 3 Department of Mechanical and Electro-Mechanical Engineering, National SunYat-sen University, Kaohsiung 80424, Taiwan; keng3@mail.nsysu.edu.tw (K.-H.L.); zhiyun@mem.nsysu.edu.tw (Z.-Y.L.); sywang@mem.nsysu.edu.tw (S.-Y.W.); hwkakaku@mem.nsysu.edu.tw (W.H.)

**Keywords:** deep learning, liver lesion segmentation, 3D segmentation display, U-Net, hepatocellular carcinoma, successive Encoder-Decoder

## Abstract

Cancer is one of the common diseases. Quantitative biomarkers extracted from standard-of-care computed tomography (CT) scan can create a robust clinical decision tool for the diagnosis of hepatocellular carcinoma (HCC). According to the current clinical methods, the situation usually accounts for high expenditure of time and resources. To improve the current clinical diagnosis and therapeutic procedure, this paper proposes a deep learning-based approach, called Successive Encoder-Decoder (SED), to assist in the automatic interpretation of liver lesion/tumor segmentation through CT images. The SED framework consists of two different encoder-decoder networks connected in series. The first network aims to remove unwanted voxels and organs and to extract liver locations from CT images. The second network uses the results of the first network to further segment the lesions. For practical purpose, the predicted lesions on individual CTs were extracted and reconstructed on 3D images. The experiments conducted on 4300 CT images and LiTS dataset demonstrate that the liver segmentation and the tumor prediction achieved 0.92 and 0.75 in Dice score, respectively, by as-proposed SED method.

## 1. Introduction

According to the health data and statistics of World Health Organization (WHO), hepatocellular carcinoma (HCC) is one of the most common cancer diseases in the world, which causes a large number of deaths every year [1]. The detection of lesions as well as the estimation of their size and number is still widely used by visual inspection of computed tomography (CT) [2] and magnetic resonance (MR) images in the clinical examination, which can be subjective. The high tumor variability generally requires reliance on the operator’s subjectivity, making it susceptible to diagnosis misinterpretations. In radiomics studies [3], all observations underline the need for automatic and reliable tools dedicated to tumor segmentation in order to finely characterize liver cancer. However, automatic segmentation [4,5,6,7,8] of liver tumor is challenging not only due to the highly variable shape of liver tumors but also because of the similar intensity values of nearby liver parenchyma.

Image segmentation is the process of dividing a digital image into multiple segments. It is a classic problem in image processing and computer vision and is widely used in medical imaging research [1,2,3,4,5,6,7,8,9,10,11,12,13,14,15,16,17,18,19,20,21,22,23,24,25,26,27,28,29,30,31,32,33,34,35,36,37,38,39,40]. In the early years, many algorithms were proposed to fulfill image segmentation. Otsu et al. [41] proposed a method based on dynamic thresholding. Vincent et al. [42] proposed a region base growing method based on computing watersheds. Those works are considered to be early studies of image segmentation.

In the medical field, Klinder et al. [9] proposed a comprehensive solution for automatically detecting and segmentation to construct compound rule-based systems for vertebra CT images. Later, machine learning (ML) [41,42,43,44,45] techniques were applied to CT images for organ segmentation. The ML-based segmentation methods did achieve superior performance than traditional ones. However, the performance of ML-based methods relies on the design of hand-crafted features, which tends to be human-defined rules, resulting in high error in the results.

In the recent years, Deep learning (DL) [5,46,47] has received increasing attention in the field of computer vision, which is an advanced branch of artificial neural network in ML. Various types of deep networks have been proposed, in which Convolutional Neural Network (CNN) [14] is one of the most popular in the image processing applications. Also, in recent years, systematic templates for DL identification processes have also emerged [48], providing researchers with a standardized rule to refer to when performing DL of such medical images. With sufficient training data, CNN can automatically learn objective and useful spatial features without any human-defined rules. Thus, CNN would be able to an appropriate solution to overcome the above-mentioned problems of tumor variability and feature design issues. Thus, the technologies and applications of CNN performing on organ/lesion segmentation would be of great interest to the medical imaging community [15,16,17,18,19,20,21,22,23,24,25,26,27,28,29,30,31,32,33,34,35,36,37,38,39,40], as this approach would have achieved superior performance in medical imaging challenges [30,31,32,33,34,35,36].

In contrast to the ML methods based on human-defined features, CNN can automatically learn discriminative features, and the learned features contain hierarchical information. Many CNN-based methods have been applied to segmentation tasks. For instance, Li et al. [16] proposed an automatic method based on 2D CNN to segment lesions from CT slices, and compared the CNN approach with other traditional ML techniques including AdaBoost [44], random forests (RF) [45], and support vector machine (SVM) [49]. The results showed that the CNN method performed significantly better than other compared methods in both qualitative and quantitative analysis. Zhang et al. [18] established a deep CNN for segmenting brain tissues on multi-modality magnetic resonance images (MRI). Lee et al. [13] proposed a CNN-based architecture for learning brain parcellation features from labelled data. However, those methods essentially perform pixel-wise classification by cropping the input. Learning of global spatial correlation is limited, which results in the failure of tumor segment with inhomogeneous densities or unclear boundaries.

To tackle this issue, other types of CNN structure have been proposed. Long et al. [15] proposed a method called Fully Convolutional Network (FCN) for semantic segmentation. FCN uses full image as input, which can further output a full resolution segmentation map with labeled colors. He et al. [20] proposed Mask-RCNN for instantaneous object segmentation, which is an extension of an object detection method called Faster-RCNN. The Mask-RCNN was then applied to uterine cervix segmentation [32]. Ünver et al. [31] combined the efficient CNN-based object detection algorithm, You Only Look Once (YOLO), with GrabCut algorithm to achieve accurate skin lesion segmentation. Ronneberger et al. [19] proposed the U-Net architecture for biomedical image segmentation. The U-Net employs two symmetric networks that performs feature extraction and segmentation map generation, respectively, which revolutionarily increases extended paths for precise targeting. This type of architecture was given a name: Encoder-Decoder (ED). Due to the dramatic segmentation performance provided by U-Net, many variants of U-Net were proposed for different applications, such as E-Net [50,51], Res-UNet [38], V-Net [23], Dense U-Net [34], One-shot Learning [52] and SD-Unet [36]. Although E-Net [50,51] has a faster training speed, there is a trade-off in terms of accuracy to improve the speed (slightly less accurate learning) by using a large Encoder and a small Decoder, there is still a difference in image detail learning compared to SegNet’s symmetric Encoder/Decoder. Further, the One-shot Learning [52] is trained by using a sample, and this kind of training with very small amount of data has excellent effect compared with other training methods.

ED has become the most popular network structure for segmentation over the past few years. Most works adopted a single ED for segmentation task for two reasons: (a) Efficient implementation and (b) end-to-end training. However, using a single ED for accurate segmentation usually requires a large amount of training data, and the performance would be sensitive to network architecture. In the case of CT imaging, the images are monochromatic and the pixel values between each tissue and organ are highly similar. Thus, this property further imposes the difficulty of the network to identify lesions in the absence of sufficient training data. Under such circumstances, a stratified strategy to partition the tumor prediction task into multiple fragments, with a single network to deal with each fragment would be a viable solution. Theoretically, this approach can reduce the difficulty of model learning and further improve the quality of segmentation.

Based on the assumption, we propose a two-stage segmentation approach, called Successive Encoder-Decoder (SED), for automatic liver tumor segmentation from CT images. The SED consists of two independent encoder-decoders, SED-1 and SED-2, which perform different segmentation tasks. The purpose of SED-1 is to localize the liver, while the purpose of SED-2 is to predict the liver lesions based on the region of interest (ROI) obtained by SED-1. More specifically, SED-1 excludes out the tissues other than liver, while SED-2 focuses on the preserved liver region to precisely extract the tumor location. SED-1 can be regarded as a pre-processing step of SED-2, which ensures that SED-2 does not segment the non-liver tissues. In terms of network composition, two different EDs for SED-1 and SED-2 were adopted, U-Net serves as the main architecture of SED-1 to localize the liver. On the other hand, tumor segmentation is a more challenging task due to irregular distribution of tumors within the liver. Thus, dense U-Net [34] was used in this project as the main network of SED-2 to achieve more efficient extual information extraction. Regarding the training of SED, the SED-1 and SED-2 must be trained independently using CT images with liver ground truths and tumor ground truths, respectively. For this purpose, we have built a liver CT dataset consisting of LiTS images [53] with a total of 4300 CT images. The experiments conducted on this dataset will demonstrate the performance on liver lesion segmentation for both quantitative and qualitative analysis. The SED segmentation results of adjacent slices as a 3D visualization image will be visualized, which is likely to assist surgeons rapidly identify the location, shape, and size of the tumors, further improving the quality of surgical treatment.

## 2. Proposed Method

### 2.1. The Overview of SED

**Figure 1:**
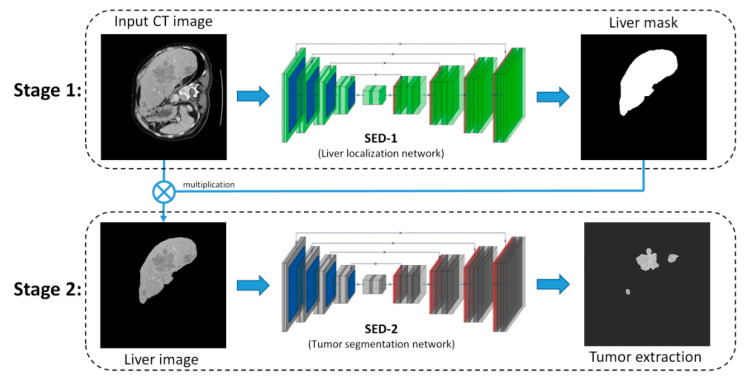
Overview of the proposed Successive Encoder-Decoder (SED) method.

As shown in Figure 1, the SED consists of two stages: Liver localization (Stage 1) and tumor extraction (Stage 2). Stage 1 uses SED-1 to exclude unwanted voxels and organs and produces a liver mask that indicates the location of liver in the CT image. Once the liver mask is obtained, the original CT image was multiplied with the mask to produce the liver image, which would be used as input for Stage 2. Then, Stage 2 uses SED-2 to extract the lesion (tumor) from the liver image. (IRB code: 201801581B0).

### 2.2. SED-1: Liver Localization Network

**Figure 2:**
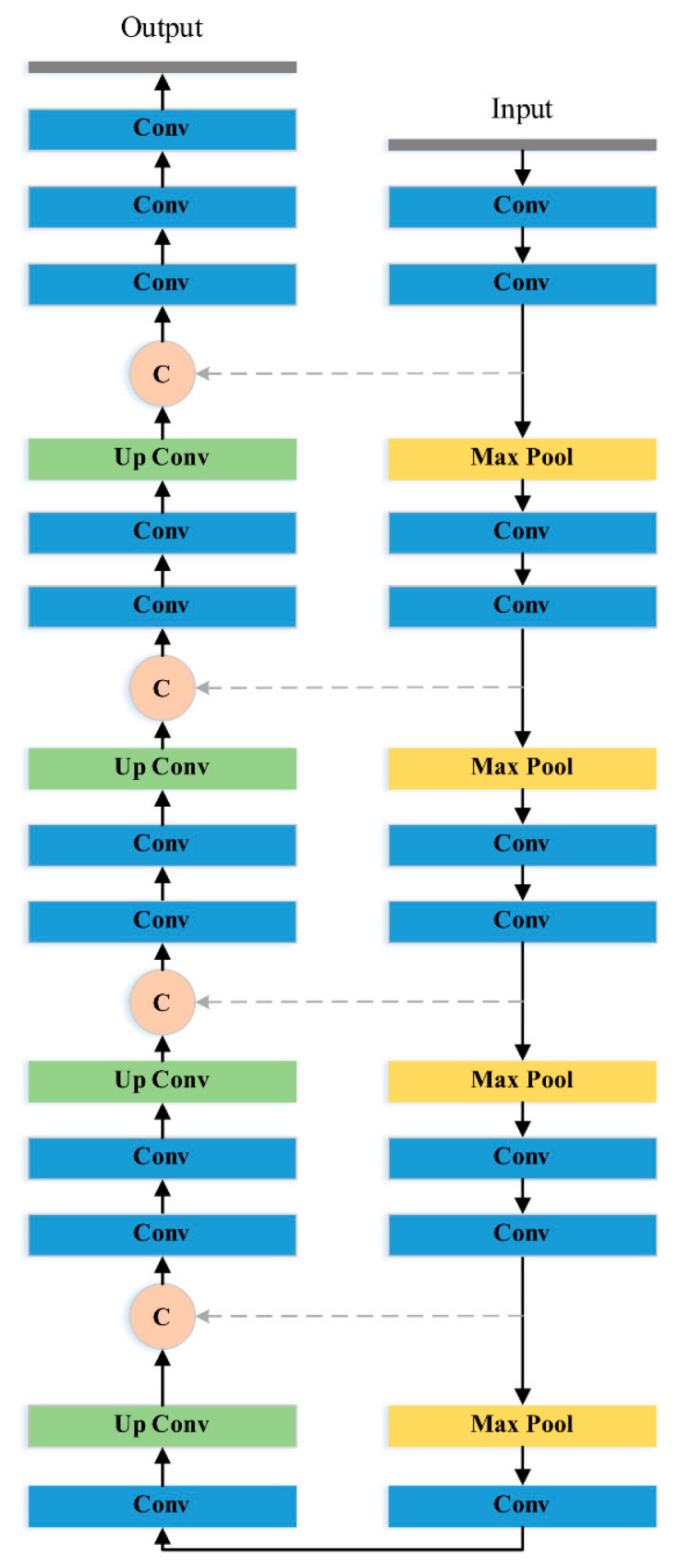
The network structure of SED-1 (U-Net) (Orange circle C means direct copy).

U-Net was adopted as the main architecture of SED-1, which is shown in Figure 2. The upper part of SED-1 is the encoder network responsible for feature extraction. The encoding process of U-Net consists of five scaling levels. Each level performs twice convolution and one pooling operation at a specific resolution. When passing through a pooling layer, a down-sampling operation is carried out to reduce image size. In order to preserve more feature information, the number of output feature maps from the convolution layer will be doubled after each down-sampling operation.

On the other hand, the lower part is the decoder network, which performs de-convolution and up-pooling. The purpose of the decoder is to restore the high-level feature maps obtained by the encoder to an output image with the same resolution of input. It is worth mentioning that the feature maps of the same level in the encoder will be concatenated to the feature maps of the up-sampling result through skip connections (gray dash lines) after each up-sampling operation. Such design can ensure that the restored feature maps contain more low-level features, thus improving the final segmentation result. Table 1 shows the definition of SED-1.

### 2.3. SED-2: Tumor Extraction Network

**Figure 3:**
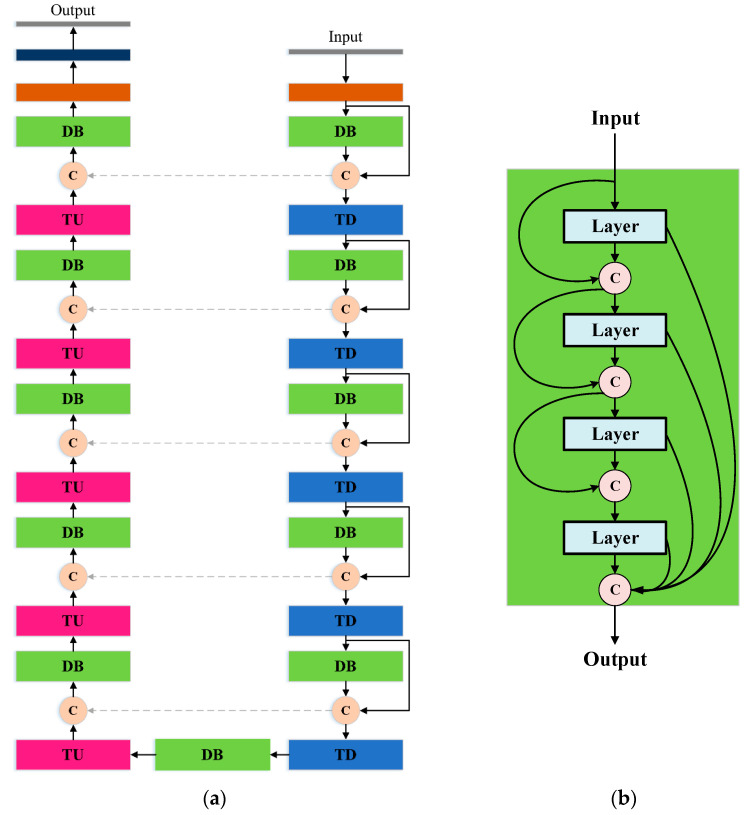
The network architecture of SED-2 (Dense U-Net): (**a**) Main architecture and (**b**) definition of dense block (DB) (Orange circle C means direct copy).

Relative to the liver area, tumors are tiny structures which are difficult to be detected due to the variability of appearances, fuzzy boundaries, uneven densities, and irregular shapes and sizes. In this case, it is required to have a more powerful encoder-decoder to localize the tumor. Therefore, the SED-2 adopted FC-DenseNet [39] (or called Dense U-Net) as the basic architecture for accurate tumor extraction. FC-DenseNet is an improved version of ED networks based on U-Net. The architecture is shown in Figure 3. As can be seen that the overall architecture consists of three types of modules: Dense block (DB), transition down (TD), and transition up (TU). DB is a core module developed in [40], which utilizes dense connections between all layers so that each layer can use feature maps of all previous layers. This design promotes feature propagation, makes features re-use efficiently, and mitigates the vanishing gradient problem. Since each layer contains the output information of all previous layers, fewer calculations of feature maps are required, thus reducing the computational complexity. As the result, the use of dense block can improve the performance of feature extraction. TD is a down-sampling operation used during the encoding process, while TU is an up-sampling operation performed by transposed convolution during the decoding process. During the decoding process, DenseNet performs feature concatenation through the skip connections (gray dashed lines) to ensure that the restored feature maps have more low-level features. Table 2 shows the architecture of SED-2.

### 2.4. Loss Function

Loss function is used to evaluate the difference between the output and the target (ground truth) in the training of deep neural network. Choosing an appropriate loss function is essential to the effectiveness of the model. In the training of SED, Dice Loss [23] was adopted as loss function to optimize the network parameters of both SED-1 and SED-2, instead of cross entropy. Dice Loss is calculated based on Dice Coefficient, which measures the similarity between two samples. The formulas of Dice Coefficient is expressed by

(1) $$\mathrm{Dice}\;\mathrm{Coefficient}=\;\frac{2\sum_{i=1}^{N}A_{i}B_{i}}{\sum_{i=1}^{N}A_{i}{}^{2}+\sum_{i=1}^{N}B_{i}{}^{2}}$$

where *A* presents the network output whose value of each pixel denotes the probability of belonging to the target, and *B* presents a binary mask (ground truth).

Dice coefficient can also be presented by $\frac{2\times \left|A\cap B\right|}{\left|A\right|+\left|B\right|}$, where |A∩B| presents the number of the intersecting pixels of *A* and *B*, and |A| and |B| presents the numbers of total pixels of *A* and *B*, respectively. The range of Dice Coefficient is [0,1]. The prediction result is more similar to the target if the value is closer to 1.

## 3. Liver CT Dataset

The CT dataset in this project consists of LiTS dataset [53], which is a publicly accessible benchmark dataset for tumor segmentation challenge. It contains 8000 CT images, which were provided by clinical institutions around the world. From the LiTS dataset, 3900 images with 512 × 512 resolution containing the ground truth of liver and tumor location were selected, which were combined with 400 Kaohsiung Chang Gung Memorial Hospital (KCGMH) images to build an experimental CT dataset. The ground truth maps of KCGMH images were annotated and plotted by KCGMH radiologists. The data provided by the hospital involved a total of three physicians who participated in the study, with the lesion section being discussed and the MASK file was created by doctors. The 4300 images were randomly split into 4000 training images and 300 test images. Referring to [10], the intensity values of all LiTS images were truncated to the range of [−100, 200] HU to remove the irrelevant details and enhance their contrast.

In order to fit the size of model as well as avoid the size limitation of GPU memory, all images were downscaled to 256 × 256 resolution to improve computational efficiency. In fact, the 256 × 256 resolution is sufficient to clearly illustrate the segmentation results of the liver and tumor.

## 4. Results and Discussion

### 4.1. Training Method, Environment, and Parameter Setting

Each training image consists of a raw CT, a liver mask, and a tumor mask. For training SED-1, the input and output are the paired data in the form of [*raw CT*, *liver mask*]. For training SED-2, the input and output are the paired data in the form of [raw CT*liver mask, *tumor mask*]. Two networks were trained independently.

For SED-1, the epoch and batch were set to 50 and 16, respectively. The initial learning rate was set to 10^−4^, and then reduced by 10% per two epochs. For SED-2, the epoch and batch were 100 and 4, respectively. The initial learning rate was also set to 10^−4^, and then multiplied by *e*^−0.9^ per two epochs (exponential decay). Both SED-1 and SED-2 adopted the ADAM optimizer for updating network parameters. Before training, 20% of the training images were randomly selected as the validation set, there is only a slight difference between testing and validation datasets. After each epoch, the model was validated once, and the model with the lowest validation loss throughout the training process is retained. Finally, the model with the lowest validation loss is designated as final model for testing.

SED training and testing were implemented on the environment: Intel^®^ Core™ i7-8700 CPU, DDR4 32 GB memory, NVIDIA GeForce 2080 Ti GPU, and Windows 10. The software was Keras 2.3.1 and Python 3.6. The training time of SED-1 and SED-2 were 1.66 and 30 h, respectively.

### 4.2. Evaluation Metrics

The performance of the proposed SED was evaluated using the following metrics: Accuracy, Intersection over Union (IoU), Similarity Coefficient (DSC), and Area Under the ROC Curve (AUC). Those metrics could be computed by four measures: *TP* (true positive), *TN* (true negative), *FP* (false positive), and *FN* (false negative). The accuracy is expressed by:

(2) $$\mathrm{Accuracy}=\frac{TP+TN}{TN+FP+TP+FN}$$

The *IoU* and *DSC* are defined by:

(3) $$\mathrm{IoU}=\frac{TP}{FP+TP+FN}$$

(4) $$\mathrm{DSC}=\;\frac{2TP}{2TP+FP+FN}$$

Finally, the AUC is obtained from a ROC curve. For each test result, a ROC curve can be created by plotting the true positive rate $\frac{TP}{TP+FN}$ against the false positive rate.$\frac{FP}{FP+TN}$ at different threshold settings. In the following section, the averaged values of four metrics of total 300 test images are reported.

### 4.3. Tumor Segmentatiuon Results

**Figure 4:**
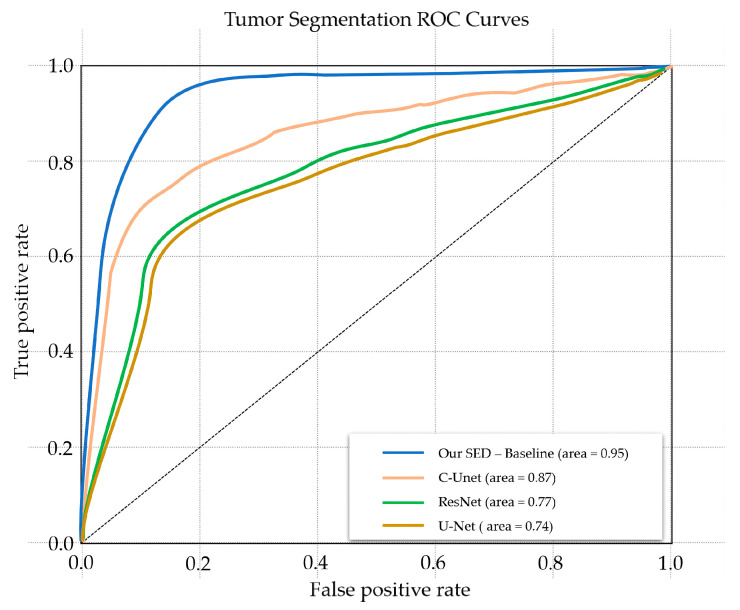
ROC curves of the state-of-art methods and the proposed SED method.

This section conducts both qualitative and quantitative studies for the proposed SED. Three state-of-art methods, U-Net [19], C-UNet [27], and ResNet [29] were selected for comparison of tumor segmentation capability. Table 3 tabulates the tumor extraction results of all the methods in ACC, IoU, DSC, and AUC. It can be seen that the proposed SED has the best overall performance in all metrics. In ACC, all methods achieved values above 0.9 due to the fact that both positive (tumor) and negative (background) samples were counted in ACC. Thus, ACC is not an ideal metric for segmentation where the positive and negative samples are unbalanced. On the contrary, IoU, DSC, and AUC were better metrics for the segmentation task. The result showed that SED significantly outperformed U-Net, ResNet, and C-UNet, corresponding to values of 0.87, 0.75, and 0.95, respectively. The ROC curves for all methods are shown in Figure 4, which were plotted by multiple pairs of (*TP*, *FP*) calculated by different threshold values.

**Figure 5:**
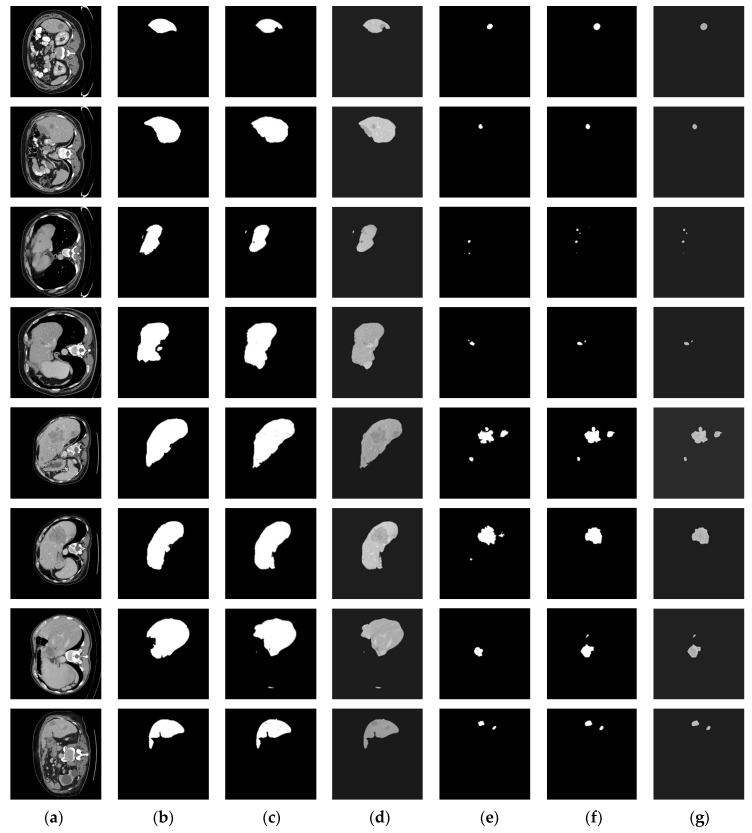
The segmentation results of the proposed SED. Each raw CT presents the result of each test image: (**a**) Raw CT image, (**b**) Liver ground truth, (**c**) SED-1 predicted liver mask, (**d**) Liver image, (**e**) Tumor ground truth, (**f**) SED-2 predicted tumor mask, and (**g**) Final tumor segmentation result.

Figure 5 shows both liver localization and tumor segmentation results of SED performed on eight selected CT samples. It can be seen that the SED provided satisfactory results for both liver localization and tumor segmentation. Compared Figure 5d with Figure 5b, SED-1 almost preserved the liver region except for those small regions where the boundaries were difficult to define. According to the results, the DSC value of SED-1 was 0.92, which implies that the liver segmentation task can be well handled using a single U-Net. In the part of tumor extraction, SED-2 also achieved ideal results. Compared Figure 5g with Figure 5e, it can be seen that most of the tumor regions were successfully captured by SED-2. Although the results indicate that the proposed SED still generate few tiny FP and FN parts, SED still provides remarkable tumor prediction capability. The contours of those parts are somehow ambiguous, low-contrast, and not clearly visible.

**Figure 6:**
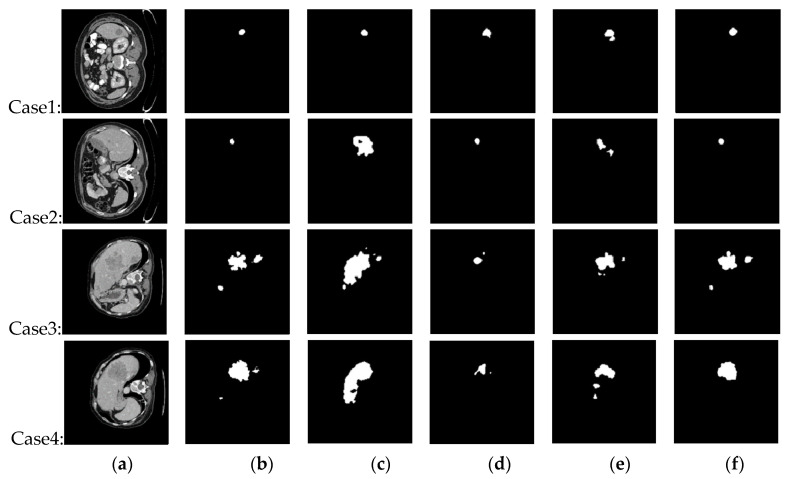
Performance comparison with state-of-art methods: (**a**) Raw CT images, (**b**) Ground truth, (**c**) U-Net [19], (**d**) ResNet [29], (**e**) C-UNet [27], and (**f**) The proposed SED.

For comparison, Figure 6 shows the tumor segmentation results of U-Net, C-UNet, ResNet, and the proposed SED. It is apparent that SED outperformed the other methods, which implies that SED utilized a two-stage stratified strategy to segment liver and tumor successively, while U-Net and ResNet are one-stage end-to-end approaches, in which the rules and features of tumor cannot be effectively learned under limited training samples. Further, it should be noted that C-UNet adopts a stratified strategy to segment the liver and tumor separately. However, the presence of FPs and FNs can be observed in Figure 6e, which indicates that the corresponding tumor segmentation results are not as good as those of SED. Conversely, the segmentation result of SED produced less segmentation errors, indicating that the primary parts of tumor were predicted more accurate.

**Figure 7:**
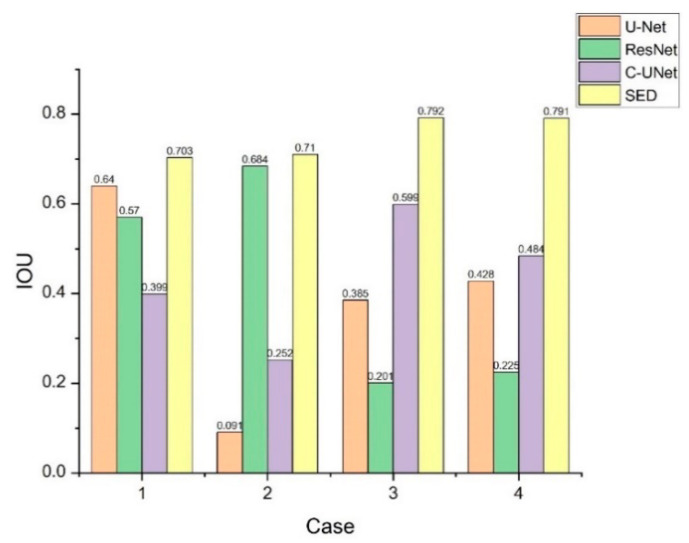
IOU value comparison from four cases by U-Net, ResNet, C-UNet, and the proposed SED.

**Figure 8:**
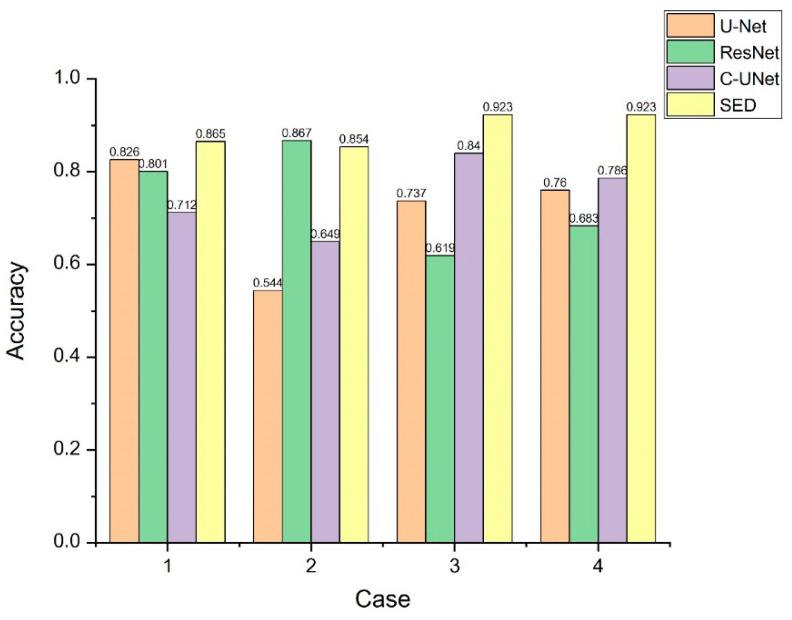
Accuracy value comparison from four cases by U-Net, ResNet, C-UNet, and the proposed SED.

Figure 7 and Figure 8 show the comparison of IOU value and accuracy for the four cases of U-Net, ResNet, C-UNet, and our proposed SED. It can be seen that our proposed SED has higher IOU values and accuracy for Case 3 and 4. Further, it can also be noted that the recognition rate of the SED model is excellent for tumor segmentation for extremely tiny particles (Case 1 and 2) and irregular shapes (Case 3). In particular, the SED model has an excellent recognition rate for the multi-sided irregular shape in Case 3 compared to U-Net where the TP portion is oversized, and to ResNet where the TP is undersized.

**Figure 9:**
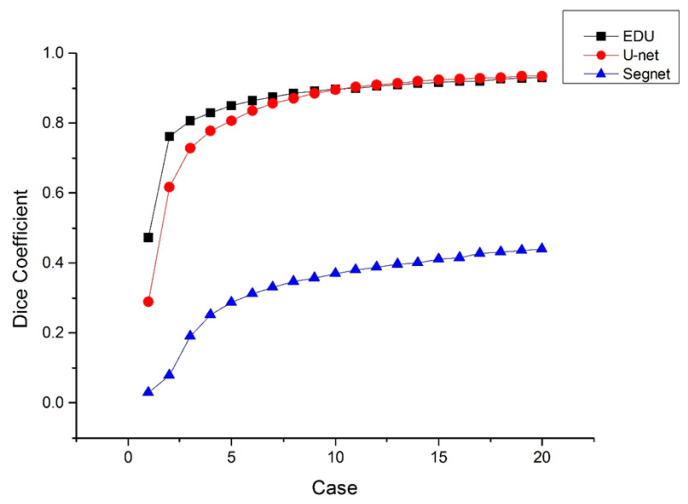
Dice Coefficient comparison from three cases by U-Net, Segnet, and EDU.

In the evaluation of the generalization capability of the model, Segnet is a well-known and widely used recognition and training module, which is designed to be efficient both in terms of memory and computational time. Further, Segnet is often used for view or larger scale recognition tools, and its architecture has more similarities to UNet, which is used as a control group. Segnet [6] and U-net with the same Encoder-Decoder framework were added to evaluate whether the two models could effectively segment the liver tumor region. Dice Score was used to compare the two models with Expanded Densely U-net, (EDU) and the result of Dice Coefficient calculated by Equation (1) is shown in Figure 9. For each model, the training Dice Score was based on the fluctuation of the training data, with approximately 20 Epochs as observation throughout the training process. The Dice Score results show a similar trend for both the EDU and U-net networks, which indicates that both EDU and U-net could predict more accurate region for tumor imaging.

The proposed SED was compared with the other five selected methods proposed in ISBI 2017 challenge [53], as shown in Table 4. In this comparison, only the LiTS dataset was used in the experiment under the same challenge rules. It can be seen that the proposed SED can achieve 0.75 in DSC while the performance of the other models achieved in the range between 0.64 and 0.7 according to the challenge report, which validates the SED.

In the field of image segmentation, image prediction is usually considered as a simple classification. However, the model does not directly output 0 or 1 predicted value classification, but outputs it into a probability graph. Thus, Sigmoid is added at the end of the model to output each category, where AUC can analyze such probability graphs. Further, AUC can be used as an indicator to judge the overall performance of the model. The more convex the curve is towards the (0, 1) point, the better the overall performance of the model. It can be simply divided as follows:

1. AUC = 0.5 (no discrimination);
2. 0.7 ≤ AUC ≤ 0.8 (acceptable discrimination);
3. 0.8 ≤ AUC ≤ 0.9 (excellent discrimination);
4. 0.9 ≤ AUC ≤ 1.0 (outstanding discrimination).

Comparison of generalization capabilities on different models is shown in Table 5. As can be seen that the performance of Segnet in ACC, IoU, DSC, and AUC is relatively poor. However, the proposed SED has a significant improvement in IoU and DSC, indicating superior generalization capabilities of SED.

**Figure 10:**
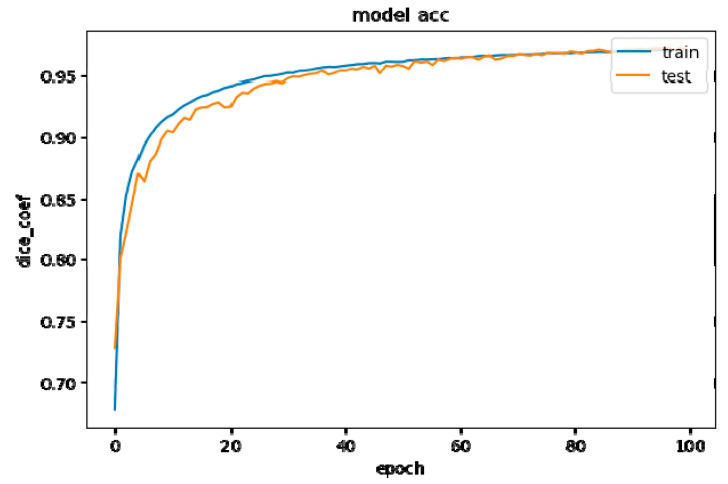
Training performance using a mix of LiTS and KCGMH with a total of 4000 images.

Training performance using a mix of LiTS and KCGMH with a total of 4000 images has been shown in Figure 10. Excellent learning results (curve fitting) were obtained after training 100 epochs with 4000 randomly mixed LiTS and KCGMH datasets. Among the 300 randomly mixed images, the result showed that an IoU value of 0.70 and an ACC value of 0.88 after eliminating the data of 5 patients with abnormal recognition due to burned liver or abnormal edema.

### 4.4. 3D Visualization

Reconstruction of three-dimensional (3D) tumor contour from two-dimensional (2D) segmentation results can be used as an alternative tool to aid clinical practice. Due to the advancement of medical imaging equipment, the slice spacing and pixels were gradually reduced, enabling the improvement of the 2D contour stitching method. In this study, Photoshop CC 2018 was used to achieve the 3D volume reconstruction of liver tumor. In the 3D reconstruction process, the 3D contours are composed of the curved surfaces formed by adjacent 2D tumor segmentation maps.

**Figure 11:**
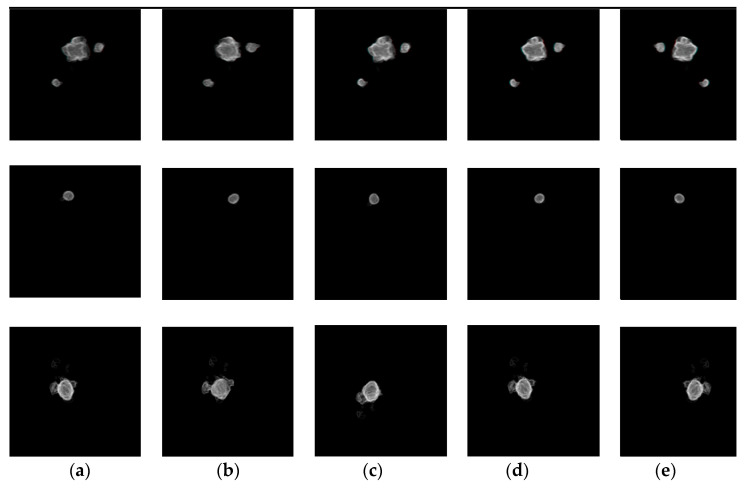
3D visualization results of three different cases. Each row shows a case of tumor segmentation results in sequential 15 slices and 3D visualization. From left to right: (**a**) Front view, (**b**) Top view, (**c**) Left view, (**d**) Bottom view, (**e**) Right view.

Figure 11 shows 3D reconstruction results of liver tumors by SED generated tumor segmentation maps. Each row presents an individual case. For each case, 15 slices were used with the interval 1mm. The result shows that 3D visualization reconstructed from the SED segmentation maps can clearly represent the size, shape, and relative position of the tumor regions, in which the volume of the tumors could be estimated. Further, the translucent 3D images facilitate the physicians’ interpretation.

**Figure 12:**
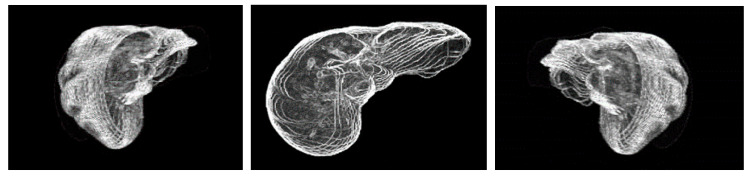
Reconstructive transparent 3D view of liver region [Rotate with the *z*-axis as the center].

Figure 12 shows the reconstructed transparent 3D view of liver region. The 3D image created by this method can be adjusted in translucency and color to create better image for a better clinical contrast. The image can be closer to reality, and further the orientation, position and size of the images can be freely adjusted. Further, the distance and location of the 3D view can be more realistic as the original DICOM image contains thickness information of each slice.

**Figure 13:**
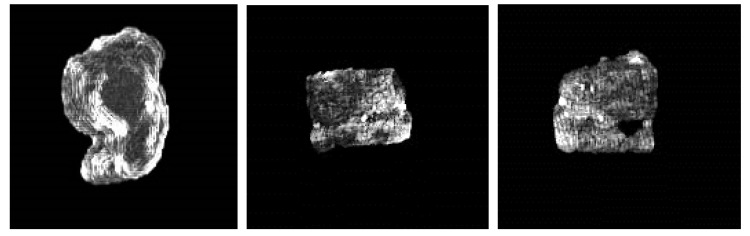
Reconstructive transparent 3D view of liver tumors [Rotate with the *z*-axis as the center].

Figure 13 illustrates the reconstructed transparent 3D view of liver tumors. The 3D image reconstructed by this proposed method can facilitate doctor to rapidly capture the size and dimension of the tumors. In addition, the reconstructed 3D tumor image can be manually rotated in any direction, position, and orientation for various perspectives. With this method, the 3D view of the tumors can be presented more concrete and specific.

## 5. Conclusions

With the advent of the era of artificial intelligence, the use of computer-based automated medicine as an aid will be one of the future trends. Using an appropriate algorithm with a computer will assist surgeons to quickly identify lesion area, reduce labor costs, and further improve medical services. Followed by this trend, many deep learning-based segmentation algorithms have been proposed for medical image processing. However, most of the existing methods only adopt a single encode-decoder (ED) as the main network architecture, which has limited performance. In this paper, a two-stage liver tumor segmentation framework, called SED, was proposed for the automatic prediction of hepatocellular carcinoma based on CT imaging. SED consists of two independent and successive encoder-decoders. The first one aims to localize the liver region through a classical ED network, while the second one performs accurate tumor segmentation through a stronger ED network. The result showed that the proposed two-stage SED method provided satisfactory liver localization and tumor segmentation performance in both quantitative and qualitative analysis, with liver segmentation and the tumor prediction reaching 0.92 and 0.75 in the Dice score, respectively. To validate the segmentation performance of the proposed SED, 4300 liver CT images composed of LiTS dataset and KCGMH dataset were conducted. Among the 300 randomly mixed LiTS and KCGMH images, the result showed that an IoU value of 0.70 and an ACC value of 0.88 after eliminating the data with abnormal recognition due to burned liver or abnormal edema. The 3D visualization images generated from the 2D segmentation results of SED could indeed provide more realistic estimates of the shape and location.

## Figures and Tables

**Table 1:** Architecture of SED-1 (Conv. stands for convolution, UP means up-sampling, [ ] denotes long range connection, [ ^ ] denotes superscript, and [ , ] denotes concatenate operation).

Encoder	Output Size	Decoder	Connecting Operation	Output Size
Input	256^2 × 1	UP 1		32^2 × 256
Conv. block 1	256^2 × 32	Copy 1	[Conv. block 4]	32^2 × 256
Pooling	128^2 × 32	Conv. block 6	[UP1, Copy1]	32^2 × 128
Conv. block 2	128^2 × 64	UP 2		64^2 × 128
Pooling	64^2 × 64	Copy 2	[Conv. block 3]	64^2 × 128
Conv. block 3	64^2 × 128	Conv. block 7	[UP2, Copy 2]	64^2 × 64
Pooling	32^2 × 128	UP 3		128^2 × 64
Conv. block 4	32^2 × 256	Copy 3	[Conv. block 2]	128^2 × 64
Pooling	16^2 × 256	Conv. block 8	[UP3, Copy 3]	128^2 × 32
Conv. block 5	16^2 × 512	UP 4		256^2 × 32
		Copy 4	[Conv. block 1]	256^2 × 32
		Conv. block 9	[UP4, Copy 4]	256^2 × 16
		Conv.		256^2 × 1

**Table 2:** Architecture of SED-2 (Conv. stands for convolution, UP means up-sampling, [ ] denotes long range connection, [ ^ ] denotes superscript, and [ , ] denotes concatenate operation).

Encoder	Output Size	Decoder	Connecting Operation	Output Size
Input	256^2 × 1	TU 1		16^2 × 240
Conv	256^2 × 48	Copy 1	[DB 5]	16^2 × 656
DB 1 (4 layers)	256^2 × 112	DB 7 (12 layers)	[TU 1, Copy 1]	16^2 × 192
TD 1	128^2 × 112	TU 2		32^2 × 192
DB 2 (5 layers)	128^2 × 192	Copy 2	[DB 4]	32^2 × 464
TD 2	64^2 × 192	DB 8 (10 layers)	[TU 2, Copy 2]	32^2 × 160
DB 3 (7 layers)	64^2 × 304	TU 3		64^2 × 160
TD 3	32^2 × 304	Copy 3	[DB 3]	64^2 × 304
DB 4 (10 layers)	32^2 × 464	DB 9 (7 layers)	[TU 3, Copy 3]	64^2 × 112
TD 4	16^2 × 464	TU 4		128^2 × 112
DB 5 (12 layers)	16^2 × 656	Copy 4	[DB 2]	128^2 × 192
TD 5	8^2 × 656	DB 10 (5 layers)	[TU 4, Copy 4]	128^2 × 80
DB 6 (15 layers)	8^2 × 880	TU 5		256^2 × 80
		Copy 5	[DB 1]	256^2 × 112
		DB 11 (4 layers)	[TU 5, Copy 5]	256^2 × 1

**Table 3:** Evaluation result by different methods.

Methods	ACC	IoU	DSC	AUC
U-Net [19]	0.92	0.53	0.65	0.73
ResNet [29]	0.98	0.62	0.67	0.77
C-UNet [27]	0.99	0.67	0.67	0.87
Our SED	0.992	0.87	0.75	0.95

**Table 4:** DSC performance comparison with five models on ISBI 2017 Challenge.

Ranking	Methods	Institution	DSC
1	Our SED	-	0.75
2	IeHealth [53]	-	0.702
3	superAI [53]	-	0.674
4	X. Han [53]	Elekta Inc.	0.67
5	E. Vorontsov et al. [53]	MILA	0.65
6	L. Bi et al. [53]	Uni Sydney	0.64

**Table 5:** Comparison of generalization capabilities on different models.

Methods	ACC	IoU	DSC	AUC
U-net [19]	0.87	0.53	0.65	0.73
ResNet [29]	0.90	0.62	0.67	0.77
C-UNet [27]	0.85	0.67	0.67	0.87
Segnet [6]	0.5	0.51	0.53	0.5
Our proposed	0.898	0.75	0.83	0.957

## Data Availability

Restrictions apply to the availability of these data. Data was obtained from Kaohsiung Chang Gung Memorial Hospital and are available H.-Y.O., C.-C.L. and Y.-F.C. with the permission of Kaohsiung Chang Gung Memorial Hospital, Taiwan.

## References

[1] Ferlay J., Shin H.R., Bray F., Forman D., Mathers C., Parkin D.M. (2010). Estimates of worldwide burden of cancer in 2008. Int. J. Cancer.

[2] Kumar V., Gu Y., Basu S., Berglund A., Eschrich S.A., Schabath M.B., Forster K., Aerts H.J., Dekker A., Fenstermacher D. (2012). Radiomics: The process and the challenges. Magn. Reson. Imaging.

[3] Echegaray S., Gevaert O., Shah R., Kamaya A., Louie J., Kothary N., Napel S. (2015). Core samples for radiomics features that are insensitive to tumor segmentation: Method and pilot study using CT images of hepatocellular carcinoma. J. Med. Imaging.

[4] Zhou Y., Xie L., Fishman E.K., Yuille A.L. Deep supervision for pancreatic cyst segmentation in abdominal CT scans. Proceedings of the International Conference on Medical Image Computing and Computer-Assisted Intervention.

[5] Shan H., Padole A., Homayounieh F., Kruger U., Khera R.D., Nitiwarangkul C., Kalra M.K., Wang G. (2018). Can deep learning outperform modern commercial CT image reconstruction methods?. Nat. Mach. Intell..

[6] Badrinarayanan V., Kendall A., Cipolla R. (2017). Segnet: A deep convolutional encoder-decoder architecture for image segmentation. IEEE Trans. Pattern Anal. Mach. Intell..

[7] Stefano A., Comelli A., Bravatà V., Barone S., Daskalovski I., Savoca G., Sabini M.G., Ippolito M., Russo G. (2020). A preliminary PET radiomics study of brain metastases using a fully automatic segmentation method. BMC Bioinform..

[8] Comelli A., Dahiya N., Stefano A., Benfante V., Gentile G., Agnese V., Raffa G.M., Pilato M., Yezzi A., Petrucci G. (2020). Deep learning approach for the segmentation of aneurysmal ascending aorta. Biomed. Eng. Lett..

[9] Klinder T., Ostermann J., Ehm M., Franz A., Kneser R., Lorenz C. (2009). Automated model-based vertebra detection, identification, and segmentation in CT images. Med. Image Anal..

[10] Varma V., Mehta N., Kumaran V., Nundy S. (2011). Indications and contraindications for liver transplantation. Int. J. Hepatol..

[11] Litjens G., Kooi T., Bejnordi B.E., Setio A.A.A., Ciompi F., Ghafoorian M., Van Der Laak J.A.M., Van Ginneken B., Sánchez C.I. (2017). A survey on deep learning in medical image analysis. Med. Image Anal..

[12] Lu R., Marziliano P., Thng C.H. Liver tumor volume estimation by semi-automatic segmentation method. Proceedings of the IEEE Engineering in Medicine and Biology 27th Annual Conference.

[13] Lee N., Laine A.F., Klein A. Towards a deep learning approach to brain parcellation. Proceedings of the IEEE International Symposium on Biomedical Imaging: From Nano to Macro.

[14] Krizhevsky A., Sutskever I., Hinton G.E. (2012). Imagenet classification with deep convolutional neural networks. Adv. Neural Inf. Process. Syst..

[15] Long J., Shelhamer E., Darrell T. Fully convolutional networks for semantic segmentation. Proceedings of the IEEE Conference on Computer Vision and Pattern Recognition.

[16] Li W., Jia F., Hu Q. (2015). Automatic segmentation of liver tumor in CT images with deep convolutional neural networks. J. Comput. Commun..

[17] Zeng Z., Xie W., Zhang Y., Lu Y.J.I.A. (2019). RIC-Unet: An Improved Neural Network Based on Unet for Nuclei Segmentation in Histology Images. IEEE Access.

[18] Zhang W., Li R., Deng H., Wang L., Lin W., Shen D. (2015). Deep convolutional neural networks for multi-modality isointense infant brain image segmentation. NeuroImage.

[19] Ronneberger O., Fischer P., Brox T. U-net: Convolutional networks for biomedical image segmentation. Proceedings of the International Conference on Medical Image Computing and Computer-Assisted Intervention.

[20] He K., Zhang X., Ren S., Sun J. Deep residual learning for image recognition. Proceedings of the IEEE Conference on Computer Vision and Pattern Recognition.

[21] SAITO K., Huimin L., Hyoungseop K., Shoji K., Tanabe M. ROI-based Fully Automated Liver Registration in Multi-phase CT Images. Proceedings of the 18th International Conference on Control, Automation and Systems (ICCAS).

[22] Hu J., Wang H., Gao S., Bao M., Liu T., Wang Y., Zhang J. (2019). S-UNet: A Bridge-Style U-Net Framework with a Saliency Mechanism for Retinal Vessel Segmentation. IEEE Access.

[23] Milletari F., Navab N., Ahmadi S.A. V-net: Fully convolutional neural networks for volumetric medical image segmentation. Proceedings of the Fourth International Conference on 3D Vision (3DV).

[24] Zheng Y.T. (2017). The Segmentation of Liver and Lesion Using Fully Convolution Neural Networks. Master’s Thesis.

[25] Kumar N., Verma R., Sharma S., Bhargava S., Vahadane A., Sethi A. (2017). A dataset and a technique for generalized nuclear segmentation for computational pathology. IEEE Trans. Med. Imaging.

[26] Havaei M., Davy A., Warde-Farley D., Biard A., Courville A., Bengio Y., Pal C., Jodoin P.M., Larochelle H. (2017). Brain tumor segmentation with deep neural networks. Med. Image Anal..

[27] Gruber N., Antholzer S., Jaschke W., Kremser C., Haltmeier M. A Joint Deep Learning Approach for Automated Liver and Tumor Segmentation. Proceedings of the 13th International conference on Sampling Theory and Applications (SampTA).

[28] Chlebus G., Meine H., Moltz J.H., Schenk A. (2017). Neural Network-Based Automatic Liver Tumor Segmentation with Random Forest-Based Candidate Filtering. arXiv.

[29] Han X. (2017). Automatic liver lesion segmentation using a deep convolutional neural network method. arXiv.

[30] Arsalan M., Owais M., Mahmood T., Cho S.W., Park K.R. (2019). Aiding the Diagnosis of Diabetic and Hypertensive Retinopathy Using Artificial Intelligence-Based Semantic Segmentation. Clin. Med..

[31] Ünver H.M., Ayan E. (2019). Skin Lesion Segmentation in Dermoscopic Images with Combination of YOLO and Grab Cut Algorithm. Diagnostics.

[32] Guo P., Xue Z., Rodney Long L., Antani S. (2020). Cross-Dataset Evaluation of Deep Learning Networks for Uterine Cervix Segmentation. Diagnostics.

[33] Benjdira B., Ouni K., Al Rahhal M.M., Albakr A., Al-Habib A., Mahrous E. (2020). Spinal Cord Segmentation in Ultrasound Medical Imagery. Appl. Sci..

[34] Kolařík M., Burget R., Uher V., Říha K., Dutta M.K. (2019). Optimized High Resolution 3D Dense-U-Net Network for Brain and Spine Segmentation. Appl. Sci..

[35] El Adoui M., Mahmoudi S.A., Larhmam M.A., Benjelloun M. (2019). MRI Breast Tumor Segmentation Using Different Encoder and Decoder CNN Architectures. Computers.

[36] Gadosey P.K., Li Y., Agyekum E.A., Zhang T., Liu Z., Yamak T., Essaf F. (2020). SD-UNet: Stripping Down U-Net for Segmentation of Biomedical Images on Platforms with Low Computational Budgets. Diagnostics.

[37] Iesmantas T., Paulauskaite-Taraseviciene A., Sutiene K. (2020). Enhancing Multi-tissue and Multi-scale Cell Nuclei Segmentation with Deep Metric Learning. Appl. Sci..

[38] Xiao X., Lian S., Zhimimg L., Li S. Weighted Res-UNet for High-quality Retina Vessel Segmentation. Proceedings of the 9th International Conference on Information Technology in Medicine and Education.

[39] Jegou S., Drozdzal M., Vazquez D., Romero A., Bengio Y. The One Hundred Layers Tiramisu: Fully Convolutional DenseNets for Semantic Segmentation. Proceedings of the IEEE Conference on Computer Vision and Pattern Recognition Workshops (CVPR).

[40] Huang G., Liu Z., Vazquez D., van der Maaten L. Densely Connected Convolutional Networks. Proceedings of the IEEE Conference on Computer Vision and Pattern Recognition Workshops (CVPR).

[41] Otsu N. (1979). A threshold selection method from gray-level histograms. IEEE Trans. Syst. Man Cybern..

[42] Vincent L., Soille P. (1991). Watersheds in digital spaces: An efficient algorithm based on immersion simulations. IEEE Trans. Pattern Anal. Mach. Intell..

[43] Bishop C.M. (2006). Pattern Recognition and Machine Learning.

[44] Collins M., Schapire R.E., Singer Y. (2002). Logistic regression, AdaBoost and Bregman distances. Mach. Learn..

[45] Breiman L. (2001). Random forests. Mach. Learn..

[46] Chollet F., Pal S. (2015). Keras.

[47] Liu W., Wang Z., Liu X., Zeng N., Liu Y., Alsaadi F.E. (2017). A survey of deep neural network architectures and their applications. Neurocomputing.

[48] Mongan J., Moy L., Kahn E.C. (2020). Checklist for Artificial Intelligence in Medical Imaging (CLAIM): A guide for authors and reviewers. Radiol. Artif. Intell..

[49] Furey T.S., Cristianini N., Duffy N., Bednarski D.W., Schummer M., Haussler D.J.B. (2000). Support vector machine classification and validation of cancer tissue samples using microarray expression data. Bioinformatics.

[50] Comelli A., Coronnello C., Dahiya N., Benfante V., Palmucci S., Basile A., Vancheri C., Russo G., Yezzi A., Stefano A. (2020). Lung Segmentation on High-Resolution Computerized Tomography Images Using Deep Learning: A Preliminary Step for Radiomics Studies. J. Imaging.

[51] Paszke A., Chaurasia A., Kim S., Culurciello E. (2016). Enet: A deep neural network architecture for real-time semantic segmentation. arXiv.

[52] Santoro A., Bartunov S., Botvinick M., Wierstra D., Lillicrap T. (2016). One-shot learning with memory-augmented neural networks. arXiv.

[53] Lits-Challenge. https://competitions.codalab.org/competitions/17094.

